# Long-term improvement of dyslipidaemia, hyperuricemia and metabolic syndrome in patients undergoing laparoscopic sleeve gastrectomy

**DOI:** 10.20945/2359-3997000000273

**Published:** 2020-06-19

**Authors:** Cornelia Zetu, Simona Popa, Andreea-Loredana Golli, Ancuta Condurache, Rubin Munteanu

**Affiliations:** 1 Department of Diabetes, Nutrition and Metabolic Diseases University of Medicine and Pharmacy “Carol Davila” Bucharest Romania Department of Diabetes, Nutrition and Metabolic Diseases, University of Medicine and Pharmacy “Carol Davila”, Bucharest, Romania; 2 Department of Diabetes, Nutrition and Metabolic Diseases University of Medicine and Pharmacy Craiova Craiova Romania Department of Diabetes, Nutrition and Metabolic Diseases, University of Medicine and Pharmacy Craiova, Craiova, Romania; 3 Department of Public Health and Healthcare Management, University of Medicine and Pharmacy Craiova Craiova Romania Department of Public Health and Healthcare Management, University of Medicine and Pharmacy Craiova, Craiova, Romania; 4 Department of Surgery Euroclinic – Regina Maria Hospital Bucharest Bucharest Romania Department of Surgery, Euroclinic – Regina Maria Hospital Bucharest, Bucharest, Romania

**Keywords:** Sleeve gastrectomy, lipid profile, metabolic syndrome, uric acid, excess BMI loss

## Abstract

**Objective:**

The aim of the study was to assess the long-term impact of laparoscopic sleeve gastrectomy (LSG) on lipid profile, uric acid level and metabolic syndrome.

**Materials and methods:**

A prospective study was performed between 2009-2014, evaluating long-term percentage of excesso body mass index loss (%EBMIL), lipid profile, uric acid level and metabolic syndrome.

**Results:**

Overall sixty subjects were followed-up. %EBMIL increased significantly, reaching a maximum (86,9 ± 6,3%) at 5 years post-LSG. Therapeutic success rate (%EBMIL ≥ 60%) was 80% at 5 years. The triglyceride level decreased significantly (148 ± 72.1 mg/dL baseline vs 130.7 ± 57.5 mg/dL at 1 month vs 110.7 ± 42.6 mg/dL at 3 months vs 92.5 ± 35.2 mg/dL at 1 year vs 84.2 ± 32.3 mg/dL at 5 years; p < 0.05 for all). HDL-cholesterol increased and uric acid decreased significantly in the first year postoperatively, remaining stable afterwards (46.9 ± 12.3 mg/dL baseline vs 47.4 ± 10 mg/dL at 1 month vs 49.8 ± 9.3 mg/dL at 3 months vs 55.4 ± 10.2 mg/dL at 1 year; p < 0.05 for all for HDL-cholesterol and 6.4 ± 2 mg/dL baseline vs 6 ± 1.7 mg/dL at 1 month vs 5.2 ± 1.3 mg/dL at 3 months vs 4.8 ± 1 mg/dL at 1 year; p < 0.05 for all for uric acid). The prevalence of metabolic syndrome decreased from 66.7% baseline to 8.3% at 5 years postoperatively (p < 0.01).

**Conclusions:**

LSG was effective in terms of %EBMIL and metabolic traits improvement for Romanian patients.

## INTRODUCTION

Obesity continues to be a major health problem because it has an ascending, rapid and important curve with epidemic proportions anywhere in the world. In the US, 66% of the population is overweight or obese. The latest estimates have shown that in the European Union, 30%-70% of the population is overweight and 10%-30% of adults are obese.

The high prevalence of obesity causes an increase in the incidence of metabolic syndrome, with an increased risk of type 2 diabetes and cardiovascular disease. Dyslipidaemia is a common feature in obese patients and a major risk factor for the development of atherosclerosis and heart-related diseases ( 1 - 5 ).

The non-pharmacological treatment of obesity, which consists of adopting a correct nutritional intake for the purpose of weight reduction along with increasing physical activity, can result in a 5%-10% reduction in body weight. Moreover, clinical research has failed to demonstrate an effect of lifestyle changes on cardiovascular morbidity and mortality.

Compared with conservative treatment, bariatric surgery is an effective long-term option in the treatment of obesity and its comorbidities through a radical effect on energy intake and carbohydrate metabolism. In support of this statement, in subjects with severe obesity, bariatric surgery results in a weight reduction of 20-30%, a reduction that is sustained over a period of at least 15-20 years.

Nowadays, bariatric surgeries are increasingly focused on lipid profile in the drive to potentially reduce cardiovascular-related diseases ( 6 , 7 ).

The objective of the current study was to assess the long-term impact of laparoscopic sleeve gastrectomy (LSG) on lipid profile, uric acid level and metabolic syndrome.

## MATERIALS AND METHODS

A prospective, observational study assessing the efficacy of LSG was performed between 2009 and 2014 in the “N.C. Paulescu” National Institute of Diabetes, Nutrition and Metabolic Diseases Bucharest and in the Clinical Hospital “Euroclinic – Regina Maria” Bucharest.

All patients that met the inclusion criteria were enrolled in the study after signing the informed consent form. All procedures were performed according to the Helsinki declaration and were approved by the Ethics Committee of University of Medicine and Pharmacy “Carol Davila” Bucharest.

Short-term (at 1 month and 3 months) and long-term (at 1 year and 5 years) clinical and biological data were evaluated in terms of percentage of excess body mass index loss (%EBMIL), percentage of excess body weight loss (%EWL), lipid profile (total cholesterol, triglycerides, HDL-cholesterol, LDL-cholesterol), uric acid level and metabolic syndrome.

%EBMIL was calculated using the formula [(pre-operative BMI − post-operative BMI)/(pre-operative BMI − 25)] × 100. Therapeutic success was defined as %EBMIL ≥ 60% ( 8 ).

%EWL was calculated using the formula [(pre-operative weight − post-operative weight)/(pre-operative weight − ideal weight)] × 100, where ideal body weight is that equivalent to a BMI of 25 kg/m^2^ ( 9 ).

Fasting plasma levels of total cholesterol, triglycerides, HDL-cholesterol and uric acid were determined using enzymatic methods. LDL-cholesterol was calculated using the Friedewald equation (LDL-cholesterol = total cholesterol − HDL-cholesterol − triglycerides/5) when plasma triglyceride concentration did not exceed 400 mg/dL.

Hypercholesterolemia was defined as total cholesterol ≥ 200 mg/dL, and hyperLDL-cholesterolemia was determined when calculated LDL-cholesterol ≥ 100 mg/dL and/or statin therapy was used. Hypertriglyceridemia was considered when triglyceride level ≥ 150 mg/dL or participants were receiving drug treatment for hypertriglyceridemia and hypoHDL-cholesterolemia when HDL-cholesterol < 40 mg/dL in men and < 50 mg/dL in women, or participants were receiving drug treatment for reduced HDL.

Hyperuricemia was considered when uric acid levels were ≥ 6 mg/dL in women or ≥ 7 mg/dL in men.

Metabolic syndrome was defined according to Join of Harmonizing the Metabolic Syndrome 2009, agreed on by the Atherosclerosis Society, International Association for the Study of Obesity, Blood Institute, American Heart Association, World Heart Federation, International Diabetes Federation Task Force on Epidemiology and Prevention, and National Heart and Lung ( 10 ).

### Surgical technique

The surgical procedure was LSG, which involves the removal of the gastric fundus and the lateral part of the gastric body (large gastric curvature) and the successive application of linear staplers that cut the stomach from the distal antrum to the Hiss angle. The residual capacity of the gastric tube was approximately 100 mL.

### Statistical analysis

The general linear model analysis for repeated measures (ANOVA) was used to test the differences in longitudinal changes of analysed parameters at each post-operative time point from baseline and from the previous one. Cochran’s test was applied to test the statistical differences for categorical variables. Spearman’s correlation analysis was used to quantify the cross-sectional relationships between variables of interest and %EBMIL. Significance level was set at p ≤ 0.05. The data were analysed using SPSS 17.0 for Windows.

## RESULTS

The study included 68 subjects, of whom 8 had incomplete data or were lost to follow-up.

The present study enrolled 60 subjects with obesity, predominantly females (83.3%, n = 50), with a mean age of 41.7 ± 12.5 years and a mean BMI of 44.6 ± 9.9 kg/m^2^. All the subjects underwent LSG.

### Therapeutic success

Defined as EBMIL ≥ 60%, therapeutic success was recorded in 71.2% of the subjects in the first year post-operatively; the proportion of subjects that achieved therapeutic success increased, reaching 80% at 5 years post-operatively ( Table 1 ). We did not find any statistically significant differences regarding the proportion of subjects with significant weight loss at 5 years *vs.* 1 year after the surgical intervention ( Table 1 ).

**Table 1:** The evolution of parameters that quantify therapeutic success and lipid parameters

	Pre-op	1 month Post-op	3 months Post-op	12 months Post-op	60 months Post-op
%EBMIL (Mean ± SD)	-	25.9 ± 3.5	56.8 ± 4.4^#^	82.9 ± 5.5^#&^	86.9 ± 6.3^#&£^
EBMIL≥60% (%)	-	5	27.6	71.2^#&^	80^#&^
Waist (cm) (Mean ± SD)	127.2 ± 19.9	118.1 ± 19.2*	107.2 ± 18.8*^#^	97.6 ± 16.4*^#&^	95.8 ± 15.8*^#&£^
Abdominal obesity (%)	100	100	89.7	84,7^#&^	88,3^#&^
Total cholesterol (mg/dL) (Mean ± SD)	217.8 ± 44.2	199.8 ± 34.8*	196.2 ± 37.4*	193.8 ± 35.7*	197.6 ± 30.8*
LDL-cholesterol (mg/dL) (Mean ± SD)	141.3 ± 44.2	126.3 ± 32.1*	124.2 ± 32.8*	119.9 ± 33.1*	122.7 ± 28.0*
Triglycerides (mg/dL) (Mean ± SD)	148.0 ± 72.1	130.7 ± 57.5*	110.7 ± 42.6*^#^	92.5 ± 35.2*^#&^	84.2 ± 32.3*^#&£^
HDL-cholesterol (mg/dL) (Mean ± SD)	46.9 ± 12.3	47.4 ± 10.0	49.8 ± 9.3	55.4 ± 10.2*^#&^	58.1 ± 12.2*^#&^
Hypercholesterolemia (%)	65	51.7	44.8	44.1	56.7
HyperLDL-cholesterolemia (%)	83.3	76.7	77.6	66.1	81.7
Hypertriglyceridemia (%)	46.7	35	19*	10.2*^#^	6.7*^#^
HypoHDL-cholesterolemia (%)	53.3	46.7	43.1	22*^#^	21.7*^#^

p < 0.05: *: 1 month. 3 months. 1 year. 5 years vs. baseline; #: 3 months. 1 year. 5 years vs. 1 month; &: 1 year. 5 years vs. 3 months; £: 5 years vs. 1 year.

The mean %EBMIL values increased significantly starting 3 months post-operatively (-56.8 ± 4.4), reaching the maximum at 5 years after the sleeve gastrectomy (-86.9 ± 6.3) ( Table 1 ). We found statistically significant differences regarding the %EBMIL values, with significant weight loss at 3 months *vs.* 1 month, at 1 year *vs.* 3 months and at 5 years *vs.* 1 year after the surgical intervention ( Table 1 ).

Subjects with pre-operative BMI < 40 kg/m^2^ had significantly higher values of %EBMIL in all evaluation stages, compared to those with BMI **≥** 40 kg/m^2^( Table 2 ), indicating that the therapeutic success was dependent on pre-operative obesity grade.

**Table 2:** The evolution of parameters that quantify therapeutic success according to BMI groups

	BMI groups	Pre-op	1 month Post-op	3 months Post-op	12 months Post-op	60 months Post-op
%EBMIL (Mean ± SD)	< 40 kg/m^2^	-	34.3 ± 14.7*	82.4 ± 43.1*	113.0 ± 50.1*	118.1 ± 50.2*
	≥ 40 kg/m^2^	-	16.9 ± 8.2	41.1 ± 11.8	63.7 ± 13.2	67.4 ± 15.5
EBMIL≥60% (%)	< 40 kg/m^2^	-	13	68.2*	95.7*	95.7*
	≥ 40 kg/m^2^	-	0	2.8	55.6	70.3

* p < 0.05 for variables between the BMI groups.

%EWL significantly increased from 19.9 ± 11.3 in the first post-operative month to 45.2 ± 16.4 after 3 months, reaching 67.1 ± 18.4 and 69.6 ± 19.4, respectively, at 1 year and 5 years follow-up (p < 0.001 for all). Abdominal circumference decreased significantly in dynamics, its decrease remaining significant even after the first year following the intervention ( Table 1 ). Waist circumference was significantly associated with %EBMIL in all evaluation stages (p < 0.001 for all).

The proportion of patients with abdominal obesity, defined as abdominal circumference ≥ 94 cm in men and ≥ 80 cm in women, decreased significantly after the third month. The percentage of subjects without abdominal obesity remained stable after the first post-operative year ( Table 1 ).

### Lipid profile

**Figure 1:**
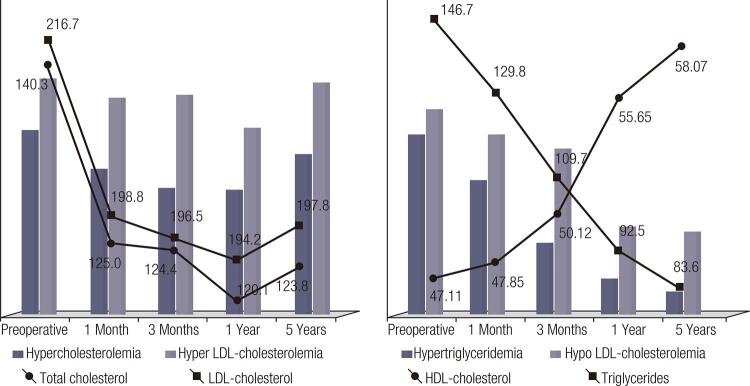
Dynamic changes of lipid profile.

The levels of total cholesterol and LDL-cholesterol progressively decreased in the first year after LSG, but the differences did not reach statistical significance. After the first year, we observed a slight increase, without statistical significance, in the total cholesterol and LDL-cholesterol ( Table 1 , Figure 1 ).

The triglyceride level decreased significantly from the first month post-operatively, the downward trend remaining significant over the duration of the study ( Table 1 , Figure 1 ).

The levels of HDL-cholesterol increased significantly, early post-operatively, the upward trend continuing during the 5 years of study ( Table 1 , Figure 1 ).

Late post-operatively, we identified a significant decrease in the percentage of patients with hypertriglyceridemia, as well as in those with abnormal HDL-cholesterol ( Table 1 , Figure 1 ). There were no significant changes in the percentages of patients presenting hypercholesterolemia or LDL-cholesterol ≥ 100 mg/dL, emphasizing the predominant influence of insulin resistance improvement on the lipid profile ( Table 1 ).

### Uric acid

**Figure 2:**
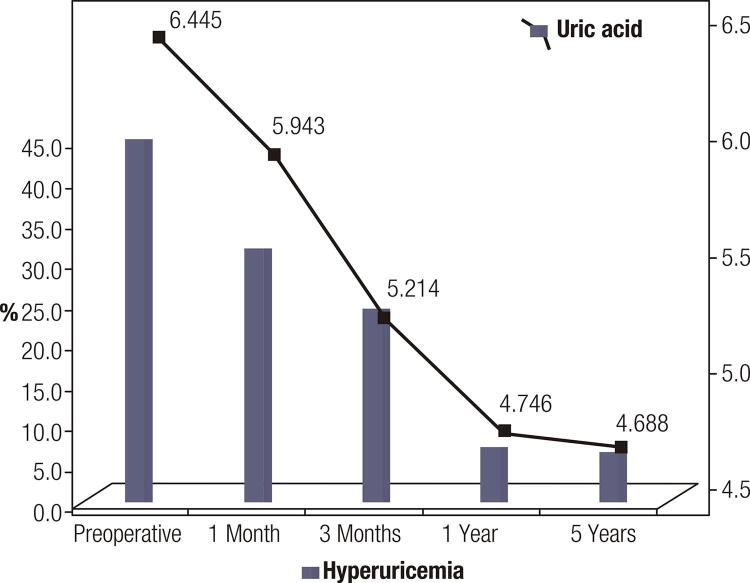
The dynamics in the changes of uric acid and in the percentage of subjects with hyperuricemia.

The percentage of subjects with chronic hyperuricemia decreased significantly after the first 3 months post-operatively, remaining stable after the first year of the study. The mean uric acid level was statistically significantly reduced early post-operatively; after the first year, however, the downward trend was not statistically significant ( Figure 2 ).

### Metabolic syndrome

The percentage of patients with metabolic syndrome decreased significantly from 66.7% at baseline to 8.3% at 5 years post-operatively. A significant decrease in the percentage of subjects presenting with an association of 4 and 5 elements of metabolic syndrome was also observed. Furthermore, at 1 year and 5 years post-operatively, no patient had an association of all 5 elements of metabolic syndrome.

**Figure 3:**
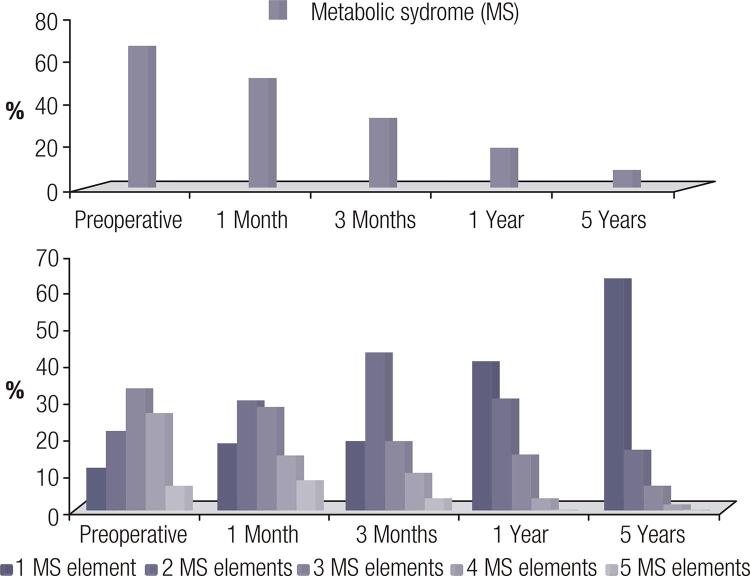
The changes in the frequency and number of metabolic syndrome elements evaluated in dynamics.

The weight loss due to surgery, as well as the post-operative improvement of all cardiometabolic parameters, explains the marked reduction in the frequency of metabolic syndrome throughout the study ( Figure 3 ).

### The correlations of pre-operative metabolic parameters and %EBMIL studied in dynamics

The pre-operative level of triglycerides was negatively correlated with %EBMIL evaluated at 1 month (p = 0.01) and 3 months (p = 0.01). Low pre-operative HDL-cholesterol was the only lipid parameter correlated with therapeutic failure evaluated at 1 year and 5 years post-operatively.

The uric acid level was significantly negatively correlated with %EBMIL evaluated at all post-operative follow-ups. Pre-operative hyperuricemia and metabolic syndrome were correlated with therapeutic failure evaluated at 3 months, 1 year and 5 years.

## DISCUSSION

### The effect of LSG on the lipid profile

The increase in HDL-cholesterol and the decrease in triglycerides are important, being two common cardiovascular risk factors associated with obesity. These results are in line with other recently published data.

A retrospective analysis by Zhang and cols. performed on 45 patients who had received LSG 1 year earlier demonstrated a significant reduction in cardiovascular risk, with an HDL-cholesterol increase of 12.7% and a decrease in triglycerides of 22.9% ( 11 ). Total cholesterol and LDL-cholesterol did not change significantly, although patients had a %EBMIL of 54% at 1 year. These data are similar to those published by Hady and cols. in 2012, where the total cholesterol concentration decreased significantly only in the first 3 months post-operatively, and then did not decrease significantly ( 12 ). Triglyceride and LDL-cholesterol values showed a statistically significant decrease at each follow-up interval (1, 3 and 6 months), 47.39% and 25.31%, respectively, at 1 year. HDL-cholesterol increased over the same period by 20.08% ( 12 ).

Vix and cols. reported a statistically significant increase in total cholesterol at 1 month and 12 months after LSG compared to the RYGB technique, with a comparable reduction in triglycerides between the two operating techniques ( 13 ).

A Spanish study published in 2013 revealed an increase in total cholesterol at 15 months after LSG, and the same level was maintained for 4 years post-operatively ( 14 ). Despite this increase, approximately 70% of patients achieved total target cholesterol at 4 years after LSG. Normal triglyceride values were recorded in 88.9% of patients and HDL-cholesterol values in 85% of cases after 4 years post-operatively.

### The effect of LSG on metabolic syndrome

In our research, the percentage of patients with metabolic syndrome significantly decreased in dynamics from 66.7% prior to surgery to 32.8%, 18.6% and 8.3% at 3 months, 1 year and 5 years post-operatively, respectively. There was a significant decrease in the percentage of subjects with association of 4 and 5 elements of metabolic syndrome; 26.7% and 6.7% of patients were observed at 1 year and 1.7% patients with 4 elements of metabolic syndrome after 5 years post-operatively, and no patients with a combination of 5 elements at 5 years after surgery.

Hady and cols. found that of 130 patients who underwent LSG bariatric surgery, pre-operatively, 56.92% had 4 criteria for the diagnosis of metabolic syndrome and 24.61% had all 5 metabolic syndrome-specific criteria ( 12 ). At 6 months post-operatively, 43.07% of the patients maintained 4 metabolic syndrome criteria and 6.15% had 5 criteria. One year after the LSG, the number of the patients with 4 criteria decreased to 21.53%, and no patient maintained all 5 metabolic syndrome criteria. An evaluation published in 2012 of the multicentre database on the effects of bariatric surgery involved approximately 23,000 patients with pre-operative metabolic syndrome ( 15 ). A metabolic syndrome resolution rate of 35% was found at 1 year after LSG.

In conclusion, LSG was an effective procedure for the patients with obesity enrolled in the study, its therapeutic success, defined as %EBMIL > 60%, being 80% at 5 years after the surgical intervention.

The results of this study prove the beneficial effects of bariatric surgery in terms of significant weight loss, with improvement of metabolic profile (improved lipid homeostasis, control of chronic hyperuricemia and metabolic syndrome) noticeable from the first month post-operatively, even before a marked weight loss was observed.
